# Clinical diagnosis and treatment of immune checkpoint inhibitor‐associated adverse events in the digestive system

**DOI:** 10.1111/1759-7714.13338

**Published:** 2020-02-28

**Authors:** Yue Li, Xiaohui Kang, Hanping Wang, Xiaoxiao Guo, Jiaxin Zhou, Lian Duan, Xiaoyan Si, Li Zhang, Xiaowei Liu, Jiaming Qian, Li Zhang

**Affiliations:** ^1^ Department of Gastroenterology, Peking Union Medical College Hospital Chinese Academy of Medicine Sciences & Peking Union Medical College Beijing China; ^2^ Department of Respiratory Medicine, Peking Union Medical College Hospital Chinese Academy of Medicine Sciences & Peking Union Medical College Beijing China; ^3^ Department of Cardiology, Peking Union Medical College Hospital Chinese Academy of Medicine Sciences & Peking Union Medical College Beijing China; ^4^ Department of Immunology, Peking Union Medical College Hospital Chinese Academy of Medicine Sciences & Peking Union Medical College Beijing China; ^5^ Department of Endocrinology, Peking Union Medical College Hospital Chinese Academy of Medicine Sciences & Peking Union Medical College Beijing China; ^6^ Department of Clinical Laboratory, Peking Union Medical College Hospital Chinese Academy of Medicine Sciences & Peking Union Medical College Beijing China; ^7^ Department of Ophthalmology, Peking Union Medical College Hospital Chinese Academy of Medicine Sciences & Peking Union Medical College Beijing China

**Keywords:** Gastrointestinal adverse events, hepatic toxicity, immune‐checkpoint inhibitors, immune‐related adverse events

## Abstract

Immunotherapy for malignant tumors is a hot spot in current research and the treatment of cancer. The activation of programmed cell death receptor‐1 (PD‐1) and cytotoxic T lymphocyte‐associated antigen 4 (CTLA)‐4 relevant signaling pathway can inhibit the activation of T lymphocytes. Tumor cells can achieve immune escape by activating this signaling pathway. By inhibiting this signaling pathway, immune‐checkpoint inhibitors (ICIs) activate T lymphocytes to clear the tumor cells. Therefore, the adverse effects of ICIs are mainly immune‐related adverse events (irAEs). The digestive system, including the gastrointestinal tract and liver which are vital organs of digestion and absorption, metabolism and detoxification, as well as important immune‐related organs, is the most commonly affected system of irAEs. This review explains the incidence, clinical features, diagnosis and treatment of liver and gastrointestinal adverse events in ICIs.

## Introduction

Checkpoint inhibitors (ICIs) can be classified into three categories based on the mechanism: (i) CTLA‐4 inibitor: for example ipilimumab, which is a monoclonal antibody targeting CTLA‐4; (ii) PD‐1 inhibitors: pembrolizumab and nivolumab are anti‐PD1 monoclonal antibodies; (iii) PD ligand 1 (PD‐L1) inhibitor: atezolizumab, avelumab and durvalumab are monoclonal antibodies which target PD‐L1. To date, the efficacy of checkpoint inhibitors have been shown in treating a variety of late‐stage malignancies including metastatic melanoma, lung cancer, renal cell cancer, hematologic malignancies and so on. ICI toxicity may cause a wide range of immune‐related adverse events (iRAEs) in any system. In this article, we review the toxicity of ICIs in the liver and gastrointestinal tract.

## Immune‐related hepatotoxicity

### Incidence

The incidence of immune‐related hepatotoxicity varies between different ICIs, mono‐ or combined therapy, and the underlying malignancy. Hepatotoxicity occurs in 5%–30% of patients receiving ICI therapy. Hepatotoxicity has been reported in up to 15% of patients receiving CTLA‐4 inhibitors, while in 5%–10% of patients with PD‐1/PDL1.^1^ Hepatitis occurred in 25%–30% (of which 15% was grade 3) of patients treated with combination therapy (ipilimumab combined with nivolumab).^2^, ^3^ The onset of hepatotoxicity has been reported to occur at any time after therapy but is most commonly seen after 8–12 weeks of treatment.^4^

### Diagnosis

The onset of hepatotoxicity in irAE is usually insidious without obvious clinical manifestation. Routinely monitoring the liver function tests before and after every cycle of treatment is crucial for early detection. The diagnosis of ICI‐related hepatotoxicy can be very challenging, since there is neither specific clinical features nor diagnostic biomarkers, and other etiologies can be coexisting such as liver malignant metastasis, infections and hepatotoxicity caused by concurrent medications.

Before ICI therapy

It is necessary to document any history of chronic liver disease including drinking habits, long‐term history of hepatotoxic medicine use, history of chronic hepatitis and autoimmune liver disease before treatment initiation. Antibodies related to autoimmune liver disease and tests for viral hepatitis including hepatitis B and hepatitis C viruses should be screened before commencing treatment.

During and after ICIs therapy

Once abnormal liver function tests have been reported, or increased compared with baseline level, a series of tests including blood biochemistry, tests for viral hepatitis, liver imaging examination and liver biopsy when needed should be completed as soon as possible. An increase in ALT or AST of more than two times the upper limit of normal should prompt workup of hepatotoxicity. Serological markers of hepatitis virus, including HAV, HBV, HCV, and opportunistic infection pathogens (CMV and EBV) should be established. Additional workup should include antinuclear antibodies (ANA), and smooth muscle antibody (SMA) tests. Medication reconciliation including evaluation for alternative therapy/herbal medication should be performed and any potential hepatotoxic medications discontinued.

Abdominal imaging

Abdominal imaging including computed tomography (CT), magnetic resonance imaging (MRI), and ultrasound (US) are usually nonspecific for the diagnosis of ICI‐related hepatitis. However, imaging evaluation can be of value in the diagnosis of metastatic liver disease and thromboembolic events. Reported imaging features of ICI‐related hepatitis include hepatomegaly, periportal edema, periportal MRI T2‐hyperintensity, attenuated liver parenchyma, and enlarged periportal lymph nodes on CT and MRI in severe hepatitis.^5^ In patients presenting with cholestasis (increased bilirubin, mainly conjugated bilirubin, and increased GGT and ALP), bile duct obstructive factors including biliary stone, tumor, or periportal lymphadenopathy should be excluded by abdominal imaging examination (ultrasound, MRCP, etc). To ensure that the diagnosis is confirmed in a timely manner and avoid delaying therapy for hepatotoxicity, identification of the above causes should be initiated early, and comprehensive evaluation before initiating ICIs is a priority.

Liver biopsy

A liver biopsy should be considered in patients with grade 4 adverse events (Table 1) to assist with a differential diagnosis and predicting prognosis. Histological examinations of ICI‐related hepatitis include nonspecific features of panlobular hepatitis indistinguishable from autoimmune hepatitis, bile duct injury including fibrin ring granulomas, central vein endotheliitis, prominent sinusoidal lymphohistiocytic infiltrates, and endotheliitis involving the central vein.^6^, ^7^ It has been reported that CTLA‐4 inhibitor‐induced hepatitis caused granulomatous hepatitis with fibrin deposits, whereas PD‐1/PD‐L1 inhibitor induced hepatitis caused lobular nongranulmatous hepatitis.^4^ A recent study has also revealed that ICI‐induced hepatitis showed increased numbers of CD3+ and CD8+ lymphocytes and decreased CD20+ B cells and CD4+ T cells compared with autoimmune hepatitis and drug‐induced liver injury.^8^

**Table 1:** Evaluation and management of hepatotoxicity of irAEs

Severity	ALT/AST	Management	Evaluation
G1	<3 × upper limit of normal (ULN)	Continue ICIs	Monitor liver function tests (LFTs) in one week
G2	3–5 × ULN	Withhold ICIs Consider prednisolone 0.5–1 mg/kg/day	Monitor LFTs every three days Discontinue concurrent medicine Limits/discontinue hepatotoxic medications (e.g., antibiotics, statins, alcohol use, etc) Rule out viral etiology, disease‐related hepatic dysfunction Consider abdominal ultrasound
G3	5–20 × ULN	Discontinue ICIs If ALT/AST < 400 and normal TBIL/INR/albumin, consider prednisolone 1 mg/kg/day If ALT/AST > 400 or abnormal TBIL/INR/albumin, initiate IV methylprednisolone 2 mg/kg/day	Evaluation as above Monitor LFTs daily Consider abdominal ultrasound Hospitalization
G4	>20 × ULN	Permanently discontinue ICIs Initiate IV methylprednisolone 2 mg/kg/day	Evaluation as above Consider hepatologist consultation and liver biopsy

ALT, alanine aminotransferase; AST, aspartate aminotransferase; ICIs, immune‐checkpoint inhibitors; LFTs, liver function tests; ULN, upper limit of normal; IV, intravenous.

### Management

The management principles of hepatotoxicity are shown in Table 1, based on the severity according to elevated level of transaminase (ALT, AST) and whether total bilirubin, INR, or albumin abnormalities were present. ICIs can usually be continued in patients confirmed to have a grade 1 hepatotoxicity. In patients with grade 2 hepatotoxicity, consideration should be given to withholding ICIs. In patients with grade 3 or Grade 4, permanent withdrawal of ICIs should be considered. Corticosteroids are the preferred therapy for treating hepatotoxicity. In patients with grade 3 or grade 4 hepatitis whose liver function tests decreased to grade 2 after IV methylprednisolone, treatment can be downgraded to oral prednisolone, and then gradually tapered within four weeks thereafter. In patients with grade 2 hepatitis who respond well to oral prednisolone, corticosteroids can be tapered within two weeks. In patients with progressively aggravated liver function during corticosteroid treatment, intensive upgraded treatment should be considered. When there is no response to intravenous methylprednisolone 2 mg/kg/day in two to three days, oral mycophenolic acid (MMF) 500–1000 mg bid should be added. Tacrolimus may be considered in case of failure after the administration of MMF. It has also been reported that antithymocyte globulin (ATG) can be used to treat severe hepatitis in patients who have failed to respond to corticosteroids.^9^ ICI‐related hepatotoxicity usually resolves within four to six weeks with the appropriate treatment. In the event that it does not resolve, it is necessary to be alerted to other contributory factors and the initial diagnostic workup should be repeated, bearing in mind the concurrent medications with potential hepatotoxicity, reactivation of CMV, as well as other causes.

## Gastrointestinal toxicity of ICIs

### Incidence

The gastrointestinal (GI) tract is the most commonly affected system with irAEs compared with other organs or systems. The incidence of GI irAE differs between different targets of ICIs. GI events caused by CTLA‐4 inhibitors are higher than that of PD‐1/PD‐L1 inhibitors. The incidence of GI irAEs also differs in patients with tumors of different origin.^10^ The incidence of GI irAEs has been reported to be higher in melanoma patients treated with PD‐1 inhibitors than that of patients with non‐small cell lung cancer (NSCLC) and renal cell cancer. It suggests that the same drug may drive the histologically specific irAE pattern in different immune microenvironments.^10^

It has been reported that diarrhea occurred in 27%–54% of cancer patients treated with CTLA‐4 inhibitors, and colitis in 8%–22%.^11^ GI adverse events are the most commonly reported irAEs of CTLA‐4 inhibitors, with the earliest onset and very severe symptoms which often leads to treatment discontinuation. It has been reported that 1%–1.5% of melanoma patients treated with ipilimumab developed colonic perforation, and that of renal cell carcinoma patients was 6.6%.^12^ The incidence of grade 3/4 GI irAEs of PD‐1 inhibitors was 1%–2%.^2^

A GI irAE may appear at any time from the first to the tenth dose of CTLA‐4 inhibitor, even several months after it was last administered.^13^ The median onset time of GI irAEs has been reported to be one month after initiation of CTLA‐4 inhibitor, and 2–4 months after PD‐1 inhibitor.^14^ GI irAEs occur more frequently, severely and early in patients with a combination treatment of CTLA‐4 inhibitors and PD‐1 inhibitors.^2^ The risk factors of GI irAEs include use of NSAIDs and medical history of inflammatory bowel disease.^13^, ^15^

### Diagnosis

Diarrhea is the most common manifestation of GI irAEs. Other symptoms include abdominal pain, blood in the stool, nausea, vomiting, weight loss, and fever. It can be accompanied by a variety of extraintestinal manifestations, such as arthralgia, endocrine disorders, skin disorders, hepatitis, nephritis, pericarditis, pancreatitis and other irAEs. Laboratory examination may show elevated levels of C‐reactive protein, anemia, and hypoalbuminemia. Positive autoimmune antibodies such as antineutrophil cytoplasmic antibody (ANCA) etc have been detected in a minority of patients.^13^ Endoscopic features are mucosal erythema, loss of vascular pattern, erosions and ulcerations. The left‐side of the colon is involved in most cases. The lesions can be diffuse or discontinuous. Histopathological features usually present as acute injury pictures (infiltration with neutrophils and eosinophils), either focal or diffuse, with crypt abscesses. In some cases, the histopathological features of chronic inflammation including disruption of crypt structures (branching, atrophy, budding, distortion, etc), basal plasmocytosis and even granuloma has been observed.^13^ There are similarities and overlaps in clinical, endoscopic and even pathological features between GI irAEs and inflammatory bowel disease (IBD), a chronic nonspecific digestive disease. Table 2 summarizes the abovementioned features and the differences compared with IBD.^16^

**Table 2:** Summary of clinical, endoscopic and pathological features of gastrointestinal irAEs

	Gastrointestinal irAEs
Clinical features	Acute onset
	Mild to life‐threatening diarrhea, bowel perforation in severe patients
	Severity of gastrointestinal differs in different ICIs
	**Higher risk in patients with a medical history of inflammatory bowel disease**
Endoscopic features	Diverse endoscopic manifestations; Rectum often spared; left colon often involved; diffuse lesion or segmental lesions
Pathological features	IBD‐like (increased basal plasma cells, crypts and apoptotic bodies are more common) Lymphocytic colitis‐like Celiac disease‐like (mostly seen in upper gastrointestinal tract) GVHD like
Immune changes	Clear immune pathogenesis
	CD4+ based T lymphocyte proliferation
	Th1/Th17 upregulation
	Interaction with intestinal microbiota

IBD, inflammatory bowel disease; ICIs, immune‐checkpoint inhibitors; GVHD, graft versus host disease.

The diagnosis of GI irAEs depends on the time relationship between the development of clinical symptoms and the administration of ICIs in patients with the above laboratory, endoscopic and histopathological features. Other causes should simultaneously be excluded, including infectious enteritis, such as *Clostridium difficile* infection, CMV colitis and other opportunistic infections; ischemic colitis; colitis caused by other medication such as NSAIDs; radiation enteritis and so on. Therefore, it is recommended to perform a stool pathogen examination including stool routine test, *Clostridium difficile* toxin, stool microscopy for leukocytes/parasites/ova, culture for drug‐resistant organisms, and viral PCR (serum CMV‐DNA). Meanwhile, an enhanced CT scan of the abdomen and pelvis, as well as sigmoidoscopy or colonoscopy with biopsy are recommended based on consultation with a gastroenterologist.

### Management

The principles of managing GI irAEs are early identification, timely and adequate treatment, and rapid escalation to improve prognosis. Treatment is stratified on the severity of diarrhea which is graded according to the frequency of bowel movements. Table 3 shows the treatment strategies of GI irAEs based on diarrhea severity. Corticosteroids are the main therapy for moderate and severe GI irAEs. In patients with a full response to corticosteroids, treatment can usually can be tapered within two to four weeks in patients with moderate GI irAEs and four to eight weeks in patients with severe symptoms. In patients with inadequate or no response to corticosteroids, dose escalation of steroids in time, and upgrading treatment to infliximab (IFX) or vedolizumab when necessary is recommended. Studies have shown that compared with long‐term steroid treatment, the therapy of short‐term steroids plus IFX reduces the risk of various opportunistic infections.^17^ Fecal microbiota transplant is reported to be valid in cases with GI irAEs refractory to steroids, IFX or vedolizumab.^18^

**Table 3:** Evaluation and management of gastrointestinal irAEs

Severity	Management	Evaluation
Mild (G1): fewer than four bowel movements per day above baseline	Continue ICIs Symptom control: hydration, loperamide Avoid high fiber/lactose diet	Stool evaluation to rule out infectious etiology: *Clostridium difficile*, CMV, etc
Moderate (G2): four to six bowel movements above baseline per day, colitis symptom (bloody diarrhea, abdominal pain)	Withhold ICIs Prednisolone 0.5–1 mg/kg/day No response in 48–72 hours, increase dose to 2 mg/kg/day	Evaluation as above GI consultation Schedule colonoscopy/sigmoidoscopy Recheck above tests every three days
Severe (G3/4): more than six bowel movements above baseline per day, other serious complications (e.g. ischemic bowel, perforation, toxic mega‐colon).	Discontinue ICIs hospitalization consider NPO, supportive care IV methylprednisolone 1‐2 mg/kg/day No response in 48 hours, continue steroids, consider adding infliximab (IFX) If IFX refractory, consider vedolizumab	Evaluation as above Consider abdominal/pelvic CT with contrast Monitor complete blood count, liver and kidney function tests, electrolytes, and C‐reactive protein every day

CMV, cytomegalovirus; ICIs, immune‐checkpoint inhibitors; IV, intravenous; IFX, infliximab; NPO, nothing by mouth.

## Intestinal microbiota, GI irAEs and tumor prognosis

Biomarkers that predict gastrointestinal irAEs have so far not been identified. Based on the latest research, fecal microbiota composition at baseline before treatment with ICIs predicts ipilimumab‐induced colitis. Studies have shown that the intestinal microbiota enriched with clostridium and other firmicutes at baseline is related to a good response to ipilimumab and higher incidence of immune‐related colitis.^19^ Several studies published in early 2018 reported that some specific fecal microbiota features prior to treatment have been associated with a good response to ICIs, which bring further prospects for enhancing the efficacy of ICIs in treating patients with cancer and improving prognosis.^20^, ^21^

## Conclusions

With the widespread use of ICIs in cancer therapy, irAEs are increasingly being valued by oncologists and specialists. The gastrointestinal system (diarrhea, colitis) is usually most affected, followed by liver involvement in digestive system irAEs. Baseline screening, early identification, timely diagnosis, rapid and adequate treatment of irAEs are key to addressing this type of clinical problem. Whether the composition of intestinal microbiota can predict GI irAEs and predict the prognosis of ICIs in treating tumors deserves further research and exploration.

## Disclosure

None of the authors has any potential financial conflict of interest related to this manuscript.
